# Real-Life Effectiveness of Smoking Cessation Delivery Modes: A Comparison Against Telephone Counseling and the Role of Individual Characteristics and Health Conditions in Quit Success

**DOI:** 10.1093/ntr/ntad195

**Published:** 2023-11-01

**Authors:** Nikita L Poole, Math J J M Candel, Marc C Willemsen, Floor A van den Brand

**Affiliations:** Department of Health Promotion, Care and Public Health Research Institute (CAPHRI), Maastricht University, Maastricht, The Netherlands; IVO Research Institute, The Hague, The Netherlands; Department of Methodology and Statistics, Care and Public Health Research Institute (CAPHRI), Maastricht University, Maastricht, The Netherlands; Department of Health Promotion, Care and Public Health Research Institute (CAPHRI), Maastricht University, Maastricht, The Netherlands; Department of Family Medicine, Care and Public Health Research Institute (CAPHRI), Maastricht University, Maastricht, The Netherlands

## Abstract

**Introduction:**

Professional behavioral counseling for smoking cessation can be delivered in many forms, which may not work equally well for everyone. We aim to explore in a real-world setting whether different delivery modes yield different rates of quit success and whether quit success varies based on gender, age, educational level, and being treated for a health condition.

**Aims and Methods:**

We used monitoring data (*n* = 13 747) from a smoking cessation counseling provider in the Netherlands (September 2018 to August 2021) to compare differences in quit success immediately after the end of counseling and at 12-month follow-up between telephone and other modes of counseling. Participants chose which mode of counseling they received. At the 12-month follow-up, we also examined differences in quit success based on demographic characteristics and whether one is being treated for various health conditions.

**Results:**

Participants of in-person group counseling and online in-company group counseling were significantly more likely to have quit immediately after the counseling compared with telephone counseling (OR = 1.25, 95% CI = 1.08–1.44; OR = 1.63, 95% CI = 1.18–2.24). Analyses revealed no significant differences in quit success between telephone and other modes of counseling after 12 months. Those treated for a respiratory or psychological condition were less likely to have maintained quit success, as were women, and participants with a lower educational level.

**Conclusions:**

When chosen by oneself, the mode of smoking cessation counseling received does not appear to be important for long-term quit success. However, certain groups warrant extra support to prevent excessive program attrition and unsuccessful quit attempts.

**Implications:**

Our findings suggest that when chosen by oneself, the delivery mode of smoking cessation counseling does not appear to be important for long-term quit success. This finding is of particular relevance for those who are unable to attend in-person cessation counseling due to, for instance, reduced accessibility or mobility. We also found that women, lower educated, and younger participants were more likely to dropout of the cessation program or to not have maintained a quit attempt, signaling that disparities in smoking cessation persist when standardized counseling is given, and therefore more tailored counseling may be necessary for these groups.

## Introduction

Professional behavioral support with pharmacotherapy for smoking cessation is promoted by national guidelines for smoking cessation.^1,2^ Behavioral support can be delivered in various forms: individually, group-based, in-person, online, or by telephone, and all can provide greater cessation rates compared with a minimal intervention such as brief advice or pharmacotherapy alone.^3–5^ However, it is important to know whether these different modes of delivery work equally well, or equally for everyone in achieving a successful, sustained quit attempt.

Evidence points to differences in smoking cessation and quit success based on individual characteristics, such as gender or having a health condition, although the literature is not definitive. Studies suggest for instance that those with COPD (chronic obstructive pulmonary disorder) have more difficulty making a successful quit attempt compared with healthy controls^6^ and that women typically have more difficulty maintaining a quit attempt compared with men.^7–9^ The literature on the influence of health conditions such as psychological disorders or diabetes is mixed.^10–17^ The evidence on the influence of age^18–21^ and educational level^19,22–24^ is far from conclusive, although there are indications that older age^20,21^ and a higher educational level^23,24^ may be associated with a greater likelihood of cessation.

The purpose of our study is to explore whether different modes of delivering real-life smoking cessation counseling are equally effective in achieving quit success and whether individual characteristics and the presence of health conditions also influence quit success. The use of real-life monitoring data provides evidence of effectiveness under real-life conditions, in which individuals choose for themselves the treatment that they will receive. The use of real-life data also allows us to examine a much larger dataset than would be typically possible in a randomized-controlled trial and provides a broader study population than is often found in clinical trials, also lending itself to sub-group comparisons. The current study is conducted in the Netherlands, where the adult smoking prevalence was 20.6% in 2021.^25^ Smoking prevalence differed by 8.1% percentage points between men and women and 8.6% percentage points between the people with the highest and lowest level of education. Smoking prevalence was the highest amongst young adults (18–24 years) (27.6%) and lowest amongst those over 65 (≤14.0%).^25^

In the current study, we compare seven different modes of smoking cessation counseling delivery with individual telephone counseling. Telephone counseling is widely used in the form of reactive quit lines and more proactive telephone-based interventions.^26^ There is moderate certainty evidence for the effectiveness of multi-session proactive telephone counseling compared with brief telephone counseling (one call) or self-help materials, and it is even more effective for those who are motivated to quit.^26^ A moderate intensity of telephone counseling (three to five calls) also appears to be more effective than one phone call.^4^ Telephone counseling can be easily tailored to the schedule and needs of the recipient and to provide heightened support in the period following a planned quit attempt.^4^ Moreover, telephone counseling is widely deployable, and many countries offer some form of telephone cessation service.^26,27^ As such, we were interested in how other delivery modes compare with proactive telephone counseling in a real-world setting in terms of achieving short- and long-term quit success.

To summarize, the research questions for the current study are (1) To what extent is quit success achieved immediately and 12 months after each mode of cessation counseling and how does this compare to proactive telephone counseling? (2) Does quit success 12 months after the cessation counseling vary for people who have various health conditions, irrespective of the type of counseling they followed? and (3) Does quit success within the different modes of cessation counseling differ based on gender, age, and educational level 12 months after cessation counseling?

## Method

### Design

Data collection took place between 5^th^ September 2018 and 20^th^ January 2022. We used data from SineFuma, a private smoking cessation counseling provider in the Netherlands. Participants were not randomized to a treatment group (delivery mode of smoking cessation counseling ), instead, they chose to enroll in one of seven delivery modes of smoking cessation counseling, as is typical in a real-life setting. Participants can enroll online or by contacting SineFuma for their counseling mode of choice. SineFuma provides neutral advice over the counseling options available, only in the case of companies offering sheltered employment if online counseling is not provided. Counseling from SineFuma is available to the general public as well as employers.

Telephone counseling was by far the most used mode of counseling. Online group counseling modes were introduced in 2020 in response to the COVID-19 pandemic. The counseling provided (“Rookvrij! Ook jij?” or “Smoke-free! You too?”) is nationally recognized as an evidence-based intervention, delivered by counselors from the KABIZ smoking cessation quality register.^28^ The content is based on national guidelines^29^ and uses the I-Change model as a theoretical background.^30^ The content across the different delivery modes is the same, however, the frequency and duration of the different modes of delivery differ (Supplementary Table S1). The third counseling session is the selected quit date. The following sessions aim to support the smoker in their quit attempt (Supplementary Table S1).

Before participation in the smoking cessation counseling, participant demographic characteristics, smoking behavior, and presence of any health conditions were measured via web-based questionnaires. Data on abstinence from smoking was recorded based on self-reporting by the smoking cessation coach of the service provider after each consultation. At the 12-month follow-up, this was recorded by the coach after a telephone call with the participant. Participants gave consent for their anonymized data to be used for research purposes.

A pre-registration protocol for this study has been published and information on the power calculations can be found (https://doi.org/10.17605/OSF.IO/8ETBM).

### Sample

For the current study, we included participants in a smoking cessation counseling by SineFuma between 1^st^ September 2018 and 31^st^ August 2021. All participants smoked cigarettes at the time of registration, were aged 15 and above, and could speak and understand Dutch. Any participants who registered but did not attend any sessions or deregistered before the start of the counseling were excluded. Participants who attended at least one session were included, if they were recorded as not attending the 3^rd^ session—the official quit date—then they were marked as having dropped out of the counseling.

Participants either approached the service provider themselves or were approached via their employer for smoking cessation counseling. They were not paid to participate in the smoking cessation counseling. Because we used monitoring data, participants were also not reimbursed for taking part in the current study. Participants could use cessation aids during the counseling. Information on their use is shown in Supplementary Table S1.

### Variables

#### Dependent Variables

The dependent variables in our study were quit success post-counseling and quit success at 12-month follow-up. The primary outcome was quit success at 12-month follow-up.

Quit success post-counseling was measured directly after the counseling ended, which is four weeks after the selected quit date. Sustained quit success was recorded by the counselor if the participant had not smoked or taken a puff of a cigarette in the last two weeks (self-report).

Quit success at the 12-month follow-up was measured on or after the 12-month mark after the quit date. Participants were contacted first via telephone and, when this was not successful, via e-mail. If they did not currently smoke, and since their quit date had not smoked at all or smoked ≤5 cigarettes, then they were counted as having achieved continued abstinence. If quit success was unknown (after three unsuccessful attempts to contact by telephone and one by e-mail) they were counted for the analysis as “abstinence not sustained.” This protocol is according to the Russel Standard (West, Hajek, Stead, & Stapleton, 2005), with the exception that self-report data was used, instead of biochemical validation of quit success.

#### Independent Variables

The independent variable was the mode of delivery of smoking cessation counseling. There were seven different delivery modes: in-person in-company group counseling, in-person group counseling for the general public, online in-company group counseling, online group counseling for the general public, individual online counseling, in-person individual counseling, and individual telephone counseling (see also Supplementary Table S1).

#### Demographic Variables

Demographic variables included were participant gender (man and woman), age, educational level, number of cigarettes smoked per day, and current treatment of health condition(s). Age was categorized into three groups: 15–39, 40–54, 55, and above; the educational level was also categorized into three groups: low (primary education and lower pre-vocational secondary education), moderate (middle pre-vocational secondary education and secondary vocational education) and high (senior general secondary education, (pre-) university education, and higher professional education).

The average number of cigarettes smoked per day was obtained by asking “*How many cigarettes do you smoke on average per day?*” This was then categorized into five groups: <10 cigarettes, 11–20 cigarettes, 21–30 cigarettes, 31+ cigarettes, and unknown.

Whether the participant was currently being treated for a health condition was measured via self-report. Participants were first asked “*Do you have/have you had* [health condition]*?*” followed by “*Are you still being treated for this?*.” If the answer to the second question given was “Yes,” then the participant was counted as currently having the health condition specified. Health conditions were subsequently grouped into five categories: cancer, cardiovascular disease, diabetes, psychological disorders (bipolar disorder, depression, eating disorder, and psychosis), and respiratory disorders (COPD and asthma).

An analysis of available cases found that a portion of the participants did not (fully) complete the online questionnaire which included questions about their educational level and the presence and treatment for any health conditions. For all respondents who partially completed this questionnaire, we treated all missing data for the questions “*Do you have/have you had [health condition]?*” followed by “*Are you still being treated for this?*” as answered “No.” All other missing values—for other variables and for respondents who had not completed the questionnaire at all—were left missing. This procedure provided 92.1% complete cases.

#### Analyses

Prior to the main analyses, we performed an attrition analysis^31^ to determine how many people had dropped out before the selected quit date and the characteristics of those participants. We also report demographic and baseline characteristics for each delivery mode of cessation counseling separately in Table 1, with differences in the number of cigarettes smoked per day, gender, age group, and education tested between participants in each delivery mode compared with telephone counseling by means of Chi-square tests.

**Table 1.** Demographic and baseline characteristics of participants in each smoking cessation counseling delivery mode, including differences in these characteristics between individual telephone counseling (TC) and all other delivery modes

				In-person counseling			Online counseling		
		Total sample	Individual telephone counseling *n* (%)	In-company group counseling *n* (%)	General group counseling *n* (%)	Individual counseling *n* (%)	In-company group counseling *n* (%)	General group counseling *n* (%)	Individual online counseling *n* (%)
Gender	Man	6532 (47.7)	2517 (46.3)	738 (58.6)	1936 (48.0)	851 (45.9)	68 (36.6)	138 (38.2)	284 (49.6)
(*n* = 13 708)	Woman	7176 (52.3)	2919 (53.7)	522 (41.4)	2101 (52.0)	1004 (54.1)	118 (63.4)	223 (61.8)	289 (50.4)
	vs TC	—	—	**χ** ^**2**^ **=** **61.638** *p* < **.001**	χ^2^**=** 2.544 *p***=** .111	χ^2^**=** 0.101 *p***=** .750	**χ** ^**2**^ **=** **6.874** *p* **=** **.009**	**χ** ^**2**^ **=** **8.893** *p* **=** **.003**	χ^2^ = 2.215 *p* = .137
Age	15–39	2918 (21.6)	1378 (25.5)	318 (27.0)	554 (13.9)	302 (16.3)	45 (24.6)	90 (24.9)	231 (40.3)
(*n* = 13 528)	40–54	4307 (32.8)	1651 (30.6)	483 (41.0)	1212 (30.4)	525 (28.3)	94 (51.4)	142 (39.3)	200 (34.9)
	55+	6303 (46.6)	2371 (43.9)	377 (32.0)	2215 (55.6)	1025 (55.3)	44 (24.0)	129 (35.7)	142 (24.8)
	vs. TC	—	—	**χ** ^**2**^ = **66.033** *p* < **.001**	**χ** ^**2**^ = **214.319** *p* < **.001**	**χ** ^**2**^ = **91.321** *p* < **.001**	**χ** ^**2**^ = **40.687** *p* < **.001**	**χ** ^**2**^ = **13.600** *p* < **.001**	**χ** ^**2**^ = **90.270** *p* < **.001**
Education	Low	4817 (42.2)	2253 (47.0)	312 (31.9)	1317 (41.2)	708 (50.0)	12 (6.9)	74 (22.6)	141 (26.2)
(*n* = 11 427)	Moderate	3641 (31.9)	1596 (33.3)	355 (36.3)	957 (29.9)	375 (26.5)	47 (26.9)	115 (35.1)	196 (36.4)
	High	2969 (26.0)	944 (19.7)	310 (31.7)	925 (28.9)	333 (23.5)	116 (66.3)	139 (42.4)	202 (37.5)
	vs. TC	—	—	**χ** ^**2**^ = **97.766** *p* < **.001**	**χ** ^**2**^ = **91.243** *p* < **.001**	**χ** ^**2**^ = **25.811** *p* < **.001**	**χ** ^**2**^ = **233.575** *p* < **.001**	**χ** ^**2**^ = **115.342** *p* < **.001**	**χ** ^**2**^ = **119.527** *p* < **.001**
Average number of cigarettes smoked^*^	<10	3435 (25.0)	1393 (25.5)	350 (27.4)	907 (22.5)	470 (25.3)	66 (35.3)	77 (21.3)	172 (30.0)
	11–20	5924 (43.1)	2375 (34.5)	569 (44.6)	1762 (43.6)	674 (36.3)	87 (46.5)	198 (54.8)	259 (45.2)
	21–30	2454 (17.9)	1037 (19.0)	127 (10.0)	786 (19.5)	328 (17.7)	25 (13.4)	55 (15.2)	96 (16.8)
	30+	623 (4.5)	270 (4.9)	20 (1.6)	206 (5.1)	87 (4.7)	0 (0.0)	14 (3.9)	26 (4.5)
(*n* = 13 747)	Unknown	1311 (9.5)	383 (7.0)	210 (16.5)	376 (9.3)	296 (16.0)	9 (4.8)	17 (4.7)	20 (3.5)
	vs. TC	—	—	**χ** ^**2**^ = **187.792** *p* < **.001**	**χ** ^**2**^ = **24.642** *p* < **.001**	**χ** ^**2**^ = **137.777** *p* < **.001**	**χ** ^**2**^ = **20.618** *p* < **.001**	**χ** ^**2**^ = **18.165** *p* = **.001**	**χ** ^**2**^ = **15.598** *p* = **.004**

^*^Cigarettes smoked daily.

Bold text indicates a significant *p* value.

Because participants may share the same counselor and, in group-based counseling, also may be part of the same group, to answer research questions 1 and 2, we conducted logistic mixed regression with random effects for counselors and, in the case of group-based counseling, also random effects for the groups. Quit success post- counseling and quit success at 12-month follow-up were the dependent variables for research question 1 and quit success at 12-month follow-up was the dependent variable for research question 2. The type of counseling was the independent variable for question 1, for which telephone counseling was taken as the reference category. Being treated or not for a specific health condition was the independent variable for research question 2. Analyses were controlled for the average number of cigarettes smoked per day, gender, age, and education level. For research question 1, we additionally controlled for those currently being treated for cancer(s), cardiovascular disease(s), diabetes, respiratory disease(s), and psychological disorder(s). For research question 2, we controlled for all medical conditions except for the medical condition of interest in the specific analyses. These analyses include all participants who completed at least the first session of the counseling. We also conducted a per-protocol analysis for research question 1 by only including participants who followed the counseling up until the selected quit date as part of their counseling, instead of all participants who participated in at least one session of the counseling. In order to control the type I error rate the Holm correction was applied.^32^

To answer research question 3, we conducted logistic mixed regression models for each mode of counseling separately, excluding the online group counseling modes due to insufficient sample sizes. Also, in this analysis random effects for counselors were included, and, in the case of group-based counseling, random effects for the groups. The dependent variable was quit success at the 12-month follow-up and the independent variables were gender, age group, and educational level. These analyses include all participants who completed at least the first session of the counseling. In order to control the type I error rate the Holm correction was applied.^32^

## Results

### Attrition Analysis

Significant associations between participants who dropped out of the counseling before the third session (and thus were not included in the per-protocol analysis) and age (χ^2^ = 28.587, *p* < .001, Cramer’s *V* < 0.01), education (χ^2^ = 15.274, *p* < .001, *V* = 0.05), and average number of cigarettes per day (χ^2^ = 170.576, *p* < .001, *V* < 0.01) were found. Participants who dropped out were significantly more likely to be aged 15–39 and less likely to be aged 55 and over (χ^2^ = 16.65, *p* < .01 and χ^2^ = 25.10, *p* < .001). They were significantly more likely to be lower educated (χ^2^ = 12.25, *p* < .001), smoke more than 30 cigarettes per day (χ^2^ = 9.67, *p* = .0019), or not report how many cigarettes they smoke on average (χ^2^ = 147.62, *p* < .001). Those who dropped out were also significantly less likely to smoke 11–20 cigarettes per day (χ^2^ = 44.76, *p* < .001).

Of participants who attended up to at least the third session, 17.5% were not able to be contacted to ascertain their smoking status at 12-month follow-up and were henceforth treated as “abstinence not sustained.” There was a significant association between dropout at follow-up and age (χ^2^ = 42.904, *p* < .001, *V* < 0.01). This group was significantly more likely to be aged 15–39 (χ^2^ = 22.09, *p* < .001) and less likely to be 55 and over (χ^2^ = 39.44, *p* < .001). There were no differences in gender, education, average number of cigarettes smoked per day, and type of training followed between the groups that could and could not be contacted at the 12-month follow-up.

### Baseline Characteristics and Differences Between Counseling Modes

Table 1 shows the baseline characteristics of the total sample and per delivery mode of counseling. The total sample comprised 52.3% women, and the largest groups in the sample were those aged 55 years or older (46.6%), those with a low level of education (42.2%), and those smoking 11–20 cigarettes per day (43.1%). The distributions of gender, age, educational level, and average number of cigarettes smoked per day, differed significantly for all delivery modes except for in-person general group counseling, in-person individual counseling, and individual online counseling when compared with telephone counseling.

### Differences in Quit Success Achieved Immediately After Each Mode of Cessation Counseling Compared with Proactive Telephone Counseling

Quit success immediately after counseling varied from 63.3 to 82.9% (Table 2). Significant differences in quit success immediately after counseling were found between telephone counseling and in-person general group counseling and between telephone counseling and online in-company group counseling (Table 3). The odds of quit success for those who followed the in-person general group counseling was 1.25 times the odds of quit success after telephone counseling (*p* = .002) and those who followed online in-company group counseling had 1.63 times the odds of quit success after telephone counseling (*p* = .003). We also conducted a secondary analysis in which only the participants were included who attended at least up to the official quit date, which yielded the same pattern of significance compared with the analyses in which all participants were included.

**Table 2.** Quit success immediately and 12 months after each mode of cessation counseling

		In-person counseling			Online counseling		
	Individual telephone counseling	In-company group counseling	General group counseling	Individual counseling	In-company group counseling	General group counseling	Individual online counseling
	% (*N*)	% (*N*)	% (*N*)	% (*N*)	% (*N*)	% (*N*)	% (*N*)
Quit success immediately after counseling	67.4 (3679)	71.4 (911)	72.6 (2932)	63.3 (1174)	82.9 (155)	72.9 (263)	67.5 (387)
Quit success 12 months after counseling (follow-up)	28.8 (1517)	31.0 (396)	30.0 (1213)	26.5 (491)	35.3 (66)	28.5 (103)	31.4 (180)
Change in percentage points	−38.6	−40.4	−42.6	−36.8	−47.6	−44.4	−36.1

**Table 3.** Differences in quit success immediately and 12 months after counseling between telephone counseling and all other types of counseling

		Quit success immediately after counseling		Quit success 12 months after counseling	
		OR (95% CI)	*p* value	OR (95% CI)	*p* value
	Individual telephone counseling	.	.	.	.
**In-person counseling**	In-company group counseling	0.96 (0.82–1.19)	*p* = .882	0.95 (0.78–1.17)	*p* = .627
	General group counseling	**1.25 (1.08–1.44)**	*p* = **.002**	0.98 (0.88–1.09)	*p* = .758
	Individual counseling	0.88 (0.74–1.04)	*p* = .135	0.91 (0.66–1.25)	*p* = .545
**Online counseling**	In-company group counseling	**1.63 (1.18–2.24)**	*p* = **.003**	1.04 (0.73–1.47)	*p* = .838
	General group counseling	1.20 (0.83–1.78)	*p* = .370	0.90 (0.66–1.21)	*p* = .474
	Individual online counseling	0.87 (0.67–1.13)	*p* = .296	1.00 (0.79–1.19)	*p* = .742

The Holm correction was applied for all six tests at T1 and for all six tests at T2 to control the type I error rate at T1 and at T2.

Bold text indicates a significant *p* value.

Analyses adjusted for the average number of cigarettes smoked per day, gender, age and educational level, and being treated for any of five health conditions.

### Differences in Quit Success Achieved 12 Months After Each Mode of Cessation Counseling Compared with Proactive Telephone Counseling

Quit success 12 months after counseling varied from 26.5 to 35.3% (Table 2). No significant differences in quit success 12 months after counseling were found between telephone counseling and any other type of counseling (Table 3). However, the confidence intervals for the three comparisons were wide (telephone counseling vs. in-person group counseling, telephone counseling vs. online in-company group counseling, and telephone counseling vs. online group counseling ). This could mean that the true difference between for instance telephone counseling and online in-company group counseling could be greater, with a potential true odds ratio (OR) of up to 1.47.

The secondary analysis, in which only the participants were included who attended at least up to the official quit date, also yielded no significant findings.

### Differences in Quit Success Achieved 12 Months After Cessation Counseling Based on Whether or not One is Being Treated for a Specific Health Condition

We tested two models as a robustness analysis (see Supplementary Table S2). In the first model, the average number of cigarettes smoked per day was included as a confounder, and since this variable could also act as a mediator, in the second model it was removed. In the first model, we saw that those being treated for a respiratory condition had 0.77 times the odds of quit success at 12 months of those not receiving treatment for a respiratory condition (95% CI = 0.68–0.88, *p* < .001). Stated otherwise, the odds of quit success at 12 months were 22.8% lower for those being treated for a respiratory condition. Likewise, the odds of quit success at 12 months for those being treated for a psychological disorder was 0.63 times the odds of quit success of those who are not being treated (95% CI = 0.54–0.72, *p* < .001). Therefore, the odds of quit success at 12 months were 37.4% lower for those being treated for a psychological disorder. Being treated for cancer or cardiovascular disease was not significantly associated with quit success at 12 months.

The second model was mostly consistent with these findings, although the ORs were slightly smaller (OR = 0.76 [95% CI = 0.67–0.87] and OR = 0.60 [95% CI = 0.52–0.70] for those being treated for a respiratory condition and psychological disorder, respectively).

### Differences in 12-Month Quit Success per Delivery Mode Based on Gender, Age, and Educational Level

Across the different delivery modes (in-person in-company counseling, in-person group counseling, in-person individual counseling, individual online counseling, and individual telephone counseling ), differences in 12-month quit success were found based on gender and educational level (Table 4). For gender, men were significantly more likely than women to have maintained quit success at 12 months for in-person group counseling.

**Table 4.** Differences in 12-month quit success per counseling delivery mode based on gender, age, and educational level (OR, 95% CI)

	In-company in-person group counseling	In-person group counseling	In-person individual counseling	Individual online counseling	Individual telephone counseling
Gender					
Man	1.13 (0.83–1.54) *p* = .449	**1.23 (1.08–1.40)** *p* = **.002**	1.26 (1.01–1.57) *p* = .037	1.58 (1.03–2.44) *p* = .039	1.12 (1.01–1.24) *p* = .033
Woman	.	.	.	.	.
Age					
15–39	0.62 (0.42–0.91) *p* = .016	0.80 (0.64–1.00) *p* = .052	0.68 (0.50–0.92) *p* = .013	0.75 (0.49–1.15) *p* = .189	0.95 (0.82–1.11) *p* = .536
40–54	0.77 (0.55–1.07) *p* = .118	0.81 (0.69–0.96) *p* = .016	0.91 (0.71–1.17) *p* = .462	0.79 (0.47–1.32) *p* = .366	0.92 (0.81–1.06) *p* = .239
55+	.	.	.	.	.
40–54 vs. 15–39	1.23 (0.84–1.80) *p* = .281	1.02 (0.78–1.33) *p* = .895	1.35 (1.03–1.76) *p* = .031	1.05 (0.72–1.54) *p* = .797	0.97 (0.84–1.11) *p* = .640
Education					
Low	0.61 (0.40–0.93) *p* = .023	**0.74 (0.63–0.88)** *p* < **.001**	**0.59 (0.46–0.760)** *p* < **.001**	0.70 (0.46–1.06) *p* = .094	**0.70 (0.61–0.80)** *p* < **.001**
Moderate	0.97 (0.680–1.39) *p* = .873	0.90 (0.75–1.07) *p* = .241	1.03 (0.86–1.23) *p* = .780	**0.53 (0.37–0.76)** *p* < **.001**	**0.76 (0.64–0.90)** *p* = **.002**
High	.	.	.	.	.
Moderate vs. low	1.59 (1.10–2.29) *p* = .014	1.22 (1.00–1.47) *p* = .046	**1.74 (1.41–2.15)** *p* < **.001**	0.76 (0.52–1.10) *p* = .142	1.08 (0.96–1.22) *p* = .213

The Holm correction was applied for all seven tests per counseling mode at to control the type I error rate.

Bold text indicates a significant *p* value.

Educational differences in 12-month quit success were found for four of the five delivery modes. Participants with a lower level of education were less likely to have maintained quit success than participants with high educational levels in three in-person delivery modes (in-person group counseling: *p* < .001; in-person individual counseling: *p* < .001 and individual telephone counseling: *p* < .001). For in-person individual counseling, they were also less likely to maintain quit success than participants with a moderate educational level (*p* < .001). For individual online counseling and individual telephone counseling, those with a moderate educational level were less likely to have maintained quit success than those with a higher level of education (*p* < .001 and *p* = .002, respectively).

## Discussion

The current study aimed to compare the real-life effectiveness of various modes of counseling delivery against individual telephone counseling. Two group-based counseling modes (in-person general group counseling and online in-company group counseling ) yielded higher quit success rates immediately after the counseling than individual telephone counseling. However, these differences were no longer present after 12 months, suggesting that the mode of delivery leads to comparable quit success in the long term when the smoker chooses the mode of delivery themselves. The disappearance of a difference in quit success between telephone and web-based counseling was also reported in a randomized trial.^33^ These findings suggest that more distant forms of counseling delivery, such as via telephone or online can yield comparable rates of quit success as in-person counseling on location, a finding of particular relevance for those with reduced mobility or accessibility to attend in-person.^34^

We found that those receiving treatment for a respiratory or psychological condition were less likely to remain abstinent 12 months after quitting. The negative association between treatment for a respiratory condition and quit success is supported by previous literature,^6^ where having COPD was associated with lower odds of quitting compared with those without comorbidities. Evidence on quit success for those with diabetes or a psychological disorder is mixed,^10–17^ however, the current study adds to the evidence that these individuals may be less likely to achieve long-term quit success. Given the higher quit attempt rates generally found in those with COPD and mental health conditions,^12^ but the lower likelihood of quit success, it is clear that quitting can be more challenging for these groups. Extra and/or tailored support when attempting to quit may be necessary, including reassurance for concerns about deteriorations in mental and physical health linked to cessation.^35,36^

Other population sub-groups that warrant extra support are younger people who smoke, smoke more cigarettes per day, women, and those with a lower educational level. Not only were these groups more likely to discontinue the counseling (except for women) but most are also often less likely to maintain quit success at 12-month follow-up. These patterns of attrition and quit success are supported by previous literature,^7,23,24,37^ with the exception of cigarettes smoked per day, which was not previously associated with attrition.^37^ Our results signal that disparities in smoking prevalence persist when standardized counseling is given, and therefore, more tailored counseling may be necessary for these groups. This includes—but is certainly not limited to—tailoring the content to discuss other issues such as stress or providing the opportunity for physical exercise.^38,39^

### Strengths and Limitations

Strengths of the research include the use of data in a real-life context and the large sample size, facilitating analysis of sub-group differences in quit success. The cessation counseling program given was consistent across the delivery modes, allowing direct comparison of the modes themselves. In addition, counseling participants were monitored up to 12 months after their quit attempt, enabling analysis of long-term quit success. A 17.5% loss to the 12-month follow-up also compares favorably to other studies.^40–43^

Quit success was self-reported rather than biochemically confirmed, which is a limitation of the study. Although agreement between self-reported and biochemically validated quit success is typically high,^44,45^ a difference as high as roughly 10% which has been previously reported could still significantly affect quit outcomes. However, differences in misreporting abstinence based on demographic characteristics or between delivery modes are unlikely to substantially vary.^46^ Due to the nature of using monitoring data from a real-world smoking cessation service, the participants were not randomly assigned to a counseling mode. This was instead chosen by the participants themselves, which can increase the risk of selection bias. In addition, due to its nonrandomized nature, the study may also be subject to confounding. To reduce this effect, we controlled the analyses for background variables such as demographic characteristics and the average number of cigarettes smoked per day. Participants were advised by trainers to be able to use pharmacological treatments alongside the counseling. However, the use of pharmacological treatments may result from self-selection to a treatment mode or through promotion during treatment on the part of the trainer, as such it could be viewed as an element of the delivery mode, lending to its effect. As such we did not control for this factor. There remains the possibility of residual confounding from variables that we were unable to control for in our analyses, such as income or use of other substances. Also, the imputation of unknown quit outcomes as unsuccessfully quit may conflate predictors of responding to follow-up with quit success. Last, the sample sizes for the online counseling modes were small, potentially influencing the precision of these results.

## Conclusions

For long-term quit success, the mode of smoking cessation counseling received does not appear to be important when chosen by people who smoke themselves. Future research should include randomization of participants to counseling modes and a comparison of counseling modes against each other or against standard care. Our study highlights sub-groups that warrant extra support to prevent excessive program attrition and unsuccessful quit attempts.

## Supplementary Material

A Contributorship Form detailing each author’s specific involvement with this content, as well as any supplementary data, are available online at https://academic.oup.com/ntr.

ntad195_suppl_Supplementary_Figure

ntad195_suppl_Supplementary_Tables

## Data Availability

The data underlying this article were provided by SineFuma B.V. by permission. Data will be shared on request to the corresponding author with permission of SineFuma B.V.
