# Supplementary material for: Real-Life Effectiveness of Smoking Cessation Delivery Modes: A Comparison Against Telephone Counseling and the Role of Individual Characteristics and Health Conditions in Quit Success
Source: Nicotine Tob Res. 2023 Nov 1;26(4):452–60. doi: 10.1093/ntr/ntad195 (PMC10959160; doi:10.1093/ntr/ntad195)
Supplement: ntad195_suppl_Supplementary_Figure [file ntad195_suppl_supplementary_figure.docx]

**Supplementary Figure 1** CONSORT diagram of participants per mode of counselling.

**Total sample** n=13747

Telephone counselling

Individual telephone counselling n=5458

In-company group counselling

n=1276

General group counselling

n=4037

Individual counselling

n=1855

In-company group counselling

n=187

General group counselling

n=361

Individual online counselling

n=573

In-person counselling

Online counselling

Smoking status unknown n=63

Smoking status unknown n=38

Smoking status unknown n=28

Smoking status unknown n=200

Smoking status unknown n=393

Smoking status unknown n=159

Smoking status unknown n=660

Analysis T2 n=343

Analysis T1 n=343

Analysis T1 n=1189

Quit success (T1)

Follow-up (T2)

Analysis T2 n=179

Analysis T1 n=179

Analysis T1 n=532

Analysis T2 n=532

Analysis T1 n=1689

Analysis T2 n=1689

Analysis T1 n=3791

Analysis T2 n=3791

Analysis T2 n=1189

Analysis T2 n=5024

Analysis T1 n=5024

Drop out n=41

Drop out n=18

Drop out n=8

Drop out n=166

Drop out n=246

Drop out n=434

Drop out n=87

Self-allocation

In the main analyses, those who discontinued the counselling before the quit date (drop out) were included and regraded as not successfully quit. In the per protocol analyses, those lost to drop out were not included (as shown in diagram). Those without smoking status data at follow-up (T2) were included and regarded as not successfully quit in both analyses.
