# Supplementary material for: Real-Life Effectiveness of Smoking Cessation Delivery Modes: A Comparison Against Telephone Counseling and the Role of Individual Characteristics and Health Conditions in Quit Success
Source: Nicotine Tob Res. 2023 Nov 1;26(4):452–60. doi: 10.1093/ntr/ntad195 (PMC10959160; doi:10.1093/ntr/ntad195)
Supplement: ntad195_suppl_Supplementary_Tables [file ntad195_suppl_supplementary_tables.docx]

**Supplementary Table 1** Description of the seven modes of delivery, cessation aid use and counselling content.

| **Delivery mode** | **Number of sessions** | **Duration per session** | **Cessation aid use†** | **Content** |
| --- | --- | --- | --- | --- |
| Individual telephone counselling | 6 | 20 minutes | 82.2% | After 2 weeks of preparation, participants make their quit attempt during the 3^rd^ meeting. They then receive another month of guidance from their appointed cessation counsellor. Topics covered include:   - The benefits of quitting smoking - How to deal with withdrawal symptoms and craving a cigarette - How to stay abstinent - Motivation - How to deal with difficult situations - How to deal with weight gain - Tips and advice, tailored to the participant’s personal situation   Contact with the cessation counsellor between appointments is possible via e-mail or telephone. Online video calls are conducted via Microsoft Teams. In the group-based delivery modes, participants are in groups of maximum 20 individuals, with an average group size of 9. |
| Group counselling |  |  |  |  |
| In-person | 7 | 1.5 hours | 85.5% |  |
| Online | 7 | 1.5 hours | 81.6% |  |
| In-company group counselling |  | |  |  |
| In-person | 7 | 1.5 hours | 70.9% |  |
| Online | 7 | 1.5 hours | 71.7% |  |
| In-person individual counselling | 6 | 1 x 30 minutes  5 x 20 minutes | 85.8% |  |
| Individual online counselling  (video calls and online support) | 6 | 20 minutes | 83.8% |  |

† Cessation aids include pharmacotherapy and e-cigarettes.

|  | **Model 1**† | | **Model 2** | |
| --- | --- | --- | --- | --- |
|  | OR (95% CI) | p-value | OR (95% CI) | p-value |
| Respiratory condition (n=2045) | **0.77 (0.68 –** **0.88)** | **p<0.001** | **0.76 (0.67 – 0.87)** | **p<0.001** |
| Diabetes (n=712) | 0.87 (0.75 – 1.00) | p=0.056 | 0.85 (0.74 – 0.98) | p=0.022 |
| Psychological disorder (n=1521) | **0.63 (0.54 –** **0.72)** | **p<0.001** | **0.60 (0.52 – 0.70)** | **p<0.001** |
| Cardiovascular disease (n=2366) | 0.99 (0.88 – 1.11) | p=0.837 | 0.98 (0.87 – 1.11) | p=0.762 |
| Cancer (n=620) | 1.12 (1.12 – 1.36) | p=0.259 | 1.11 (0.91 – 1.34) | p=0.313 |

**Supplementary Table 2** Differences in quit success 12-months after smoking cessation counselling for respondents being treated for various health conditions.

† Model 1: adjusted for the average number of cigarettes smoked per day, gender, age and educational level. Model 2: adjusted for gender, age and educational level.

The Holm correction was applied for all five tests per counselling mode at to control the type I error rate. Bold text indicates a significant p-value, the reference group is the group not being treated for the condition.
